# Supplementary material for: Efficacy of systemic temozolomide‐activated phage‐targeted gene therapy in human glioblastoma
Source: EMBO Mol Med. 2019 Feb 27;11(4):e8492. doi: 10.15252/emmm.201708492 (PMC6460351; doi:10.15252/emmm.201708492)
Supplement: Supplementary file 1 — Expanded View Figures PDF [file EMMM-11-e8492-s001.pdf]

Expanded View Figures

A

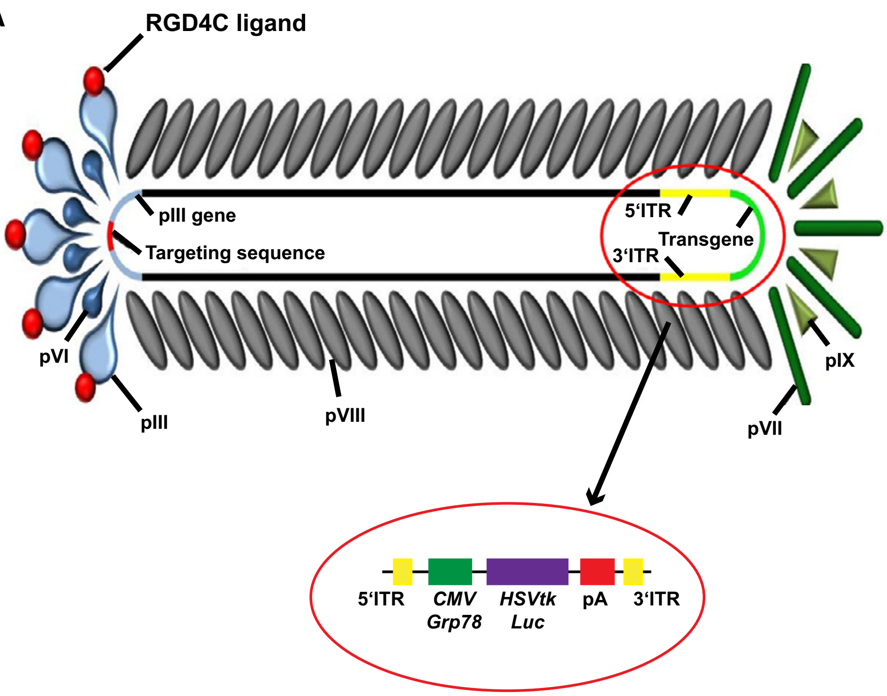

B

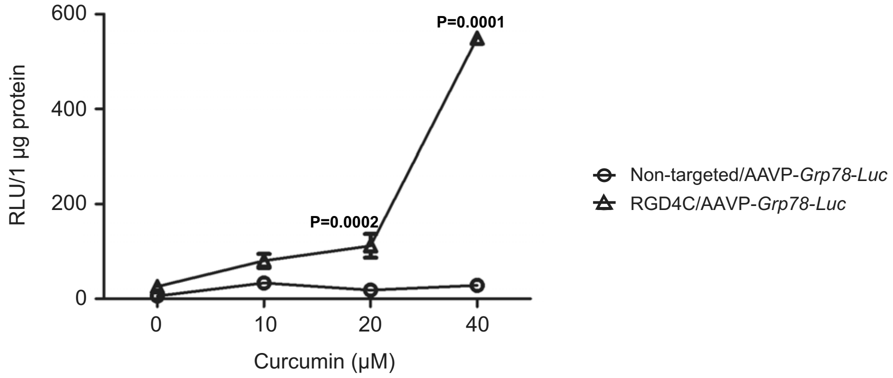

Figure EV1. The targeted RGD4C/AAVP viral particle.

A The vector bears the $\alpha_v\beta_3$ integrin-targeting double-cyclic RGD4C ligand on the pIII minor coat protein. The virus structure consists of 2,700–3,000 copies of the major coat protein pVIII with approximately five copies of the four minor capsid proteins pIII, pVI, pVII, and pIX, which are located at the ends of the filamentous particle. The AAV transgene cassette flanked by the inverted terminal repeats (ITR) from AAV2 is inserted in an intergenomic region of the bacteriophage genome. Expression of the *HSVtk* or *Luc* transgenes is under the control of either *CMV* or *Grp78* promoters. pA: polyadenylation signal.

B Induction of RGD4C/AAVP-*Grp78* by curcumin in primary glioma. Pediatric human primary glioma cells transduced with RGD4C/AAVP-*Grp78-Luc* or non-targeted/AAVP-*Grp78-Luc* control vector were treated with curcumin at day 3 post-transduction. Results represent the RLU measured at day 6 post-transduction and normalized to untreated and non-transduced control cells. Data shown are representative of three independent experiments, $n = 3$. Data analysis was done by two-way ANOVA and Bonferroni's multiple comparison test.

Data information: Data are expressed as mean ± SEM.
Source data are available online for this figure.

© 2019 The Authors

Figure EV2. Toxicity evaluation following treatments of nude mice with intracranial GBM.

A Weights of nude mice bearing intracranial U87 tumors, from all experimental groups, before and after treatment. Data shown are representative of two experiments, $n = 5$.

B Weight of nude mice with intracranial human primary HSJD-GBM-001 throughout the experiment, from all experimental groups. Data shown are representative of two experiments, $n = 5$.

C H&E staining of the healthy tissues recovered at day 12 following initiation of treatments of HSJD-GBM-001-bearing mice. Scale bar, 100 μm.

D Immunostaining analysis of phage in normal tissues and primary HSJD-GBM-001 after intravenous administration of RGD4C/AAVP-*HSVtk* vector (5×10^{10} TU) into nude mice bearing HSJD-GBM-001 derived tumors. Arrows indicate phage staining. The low-magnification inserts represent control staining of the tissue sections with the secondary antibody alone. Scale bars, 100 μm.

Source data are available online for this figure.

© 2019 The Authors

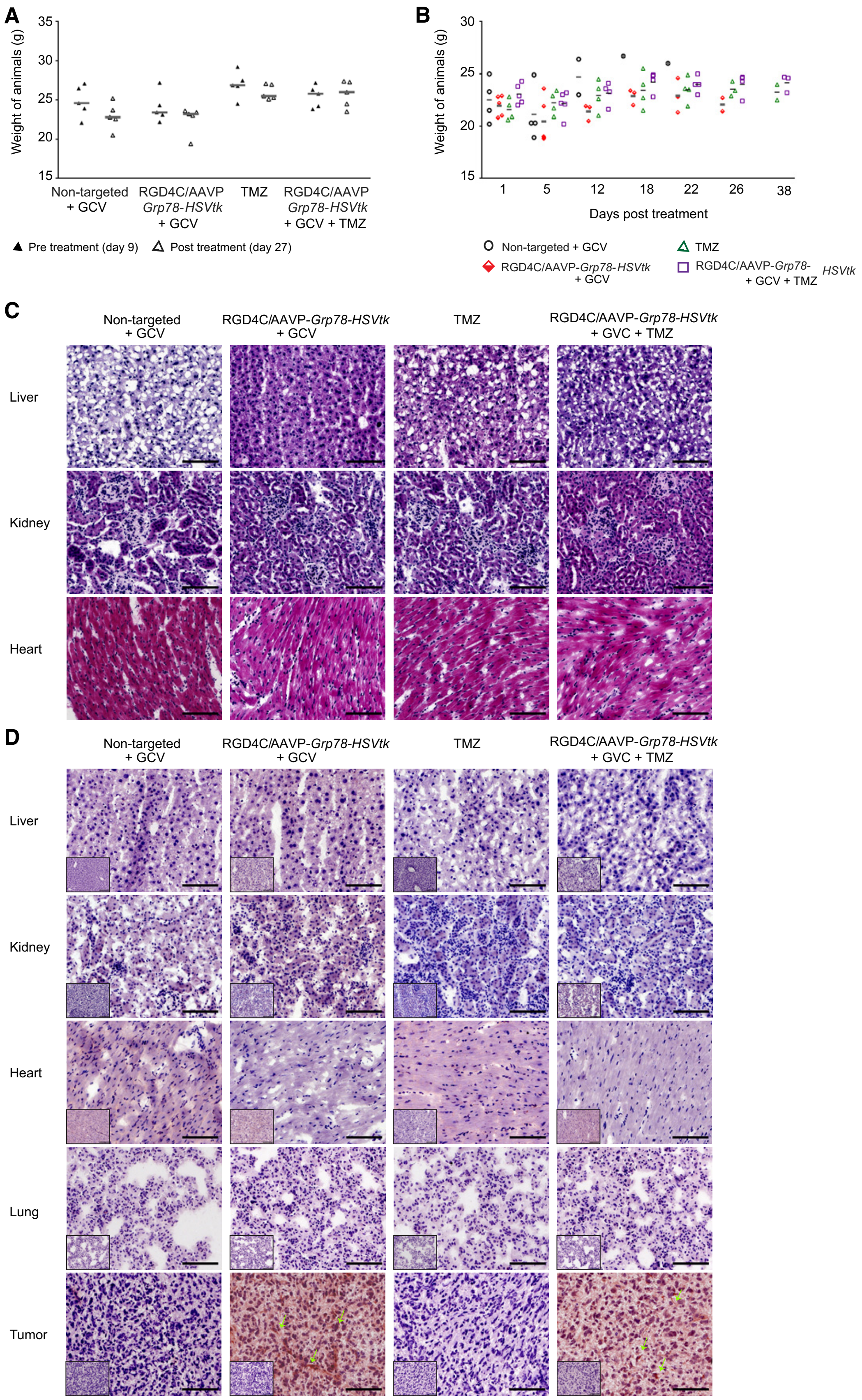

Figure EV2.

© 2019 The Authors

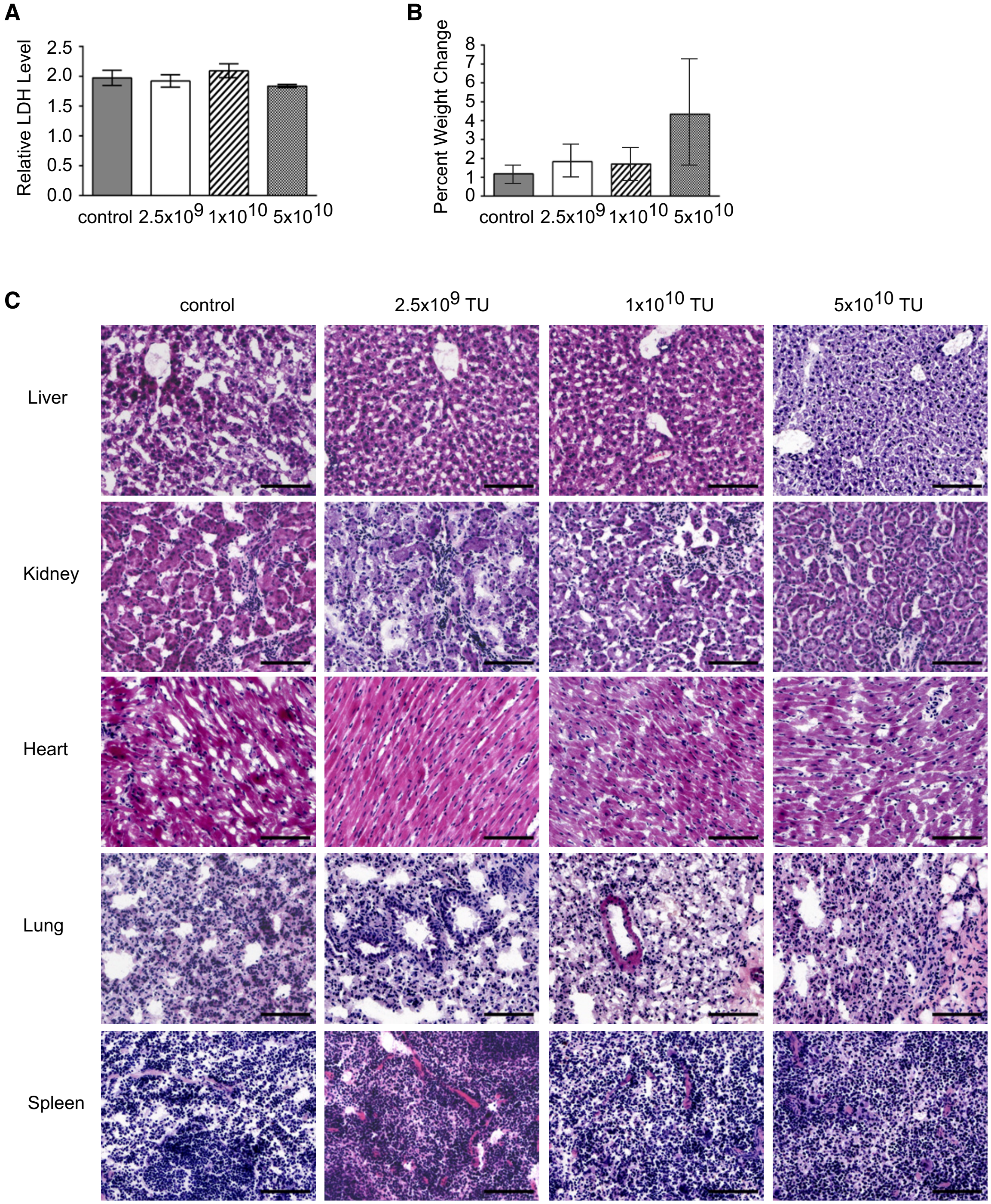

Figure EV3. Toxicity study in wild-type mice.

Mice, female BALB/c, were administered intravenously with increasing doses of the RGD4C/AAVP-*Gp78-HSVtk* as follows: 2.5×10^9 TU (1×10^{11} TU/kg), 1×10^{10} TU (5×10^{11} TU/kg), or 5×10^{10} TU (2×10^{12} TU/kg), a control group of mice received vehicle and no phage, then tissues and sera collected at day 7 post-vector administration.

A Evaluation of LDH production in the sera of all animals. Data shown are representative of two experiments (n = 6 for each vector dose).
B Weight change of animals measured over 7 days following vector delivery. Data shown are representative of two experiments (n = 6 for each vector dose).
C Histopathological analysis of the healthy tissues. Scale bar, 100 μm.

Data information: Data are expressed as mean ± SEM.
Source data are available online for this figure.

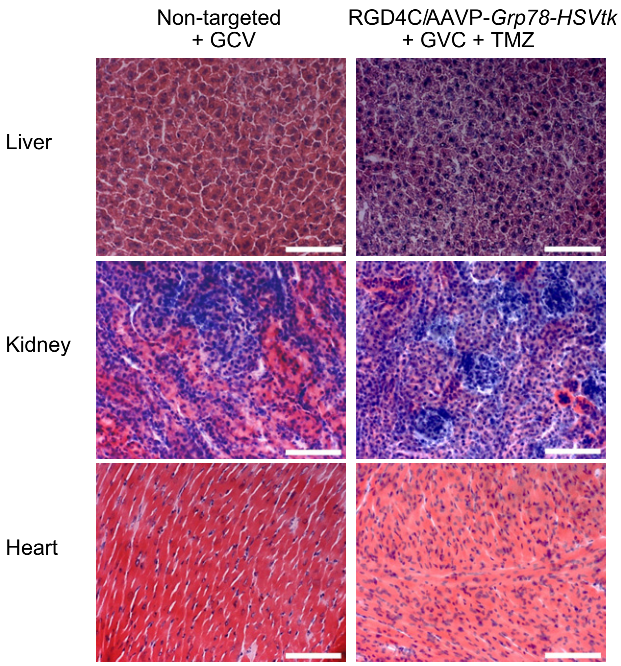

Figure EV4. Histological analysis of healthy tissues in immunocompetent mice.

H&E staining of liver, kidney, and heart recovered after therapy from the C57BL/6J mice. Scale bar, 100 μm.

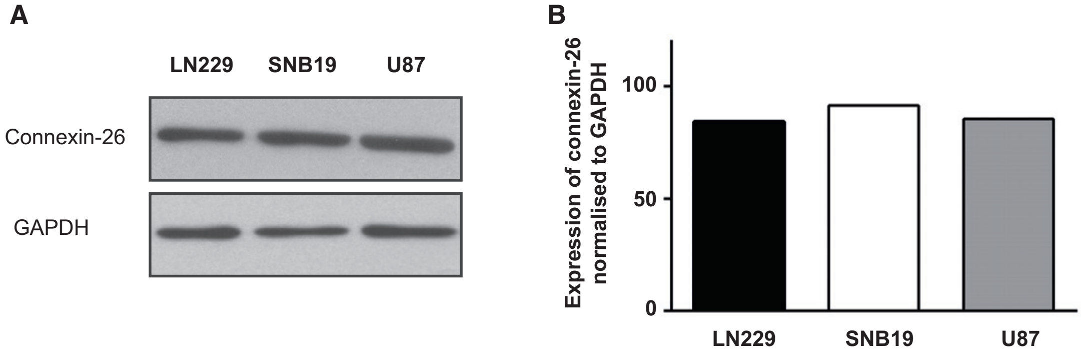

Figure EV5. Connexin-26 expression in LN229, U87, and SNB19 cell lines.

A Western blot using antibodies to connexin-26 and GAPDH.
B Quantification of connexin-26 protein levels.

Source data are available online for this figure.

 © 2019 The Authors
