# Supplementary material for: Efficacy of systemic temozolomide‐activated phage‐targeted gene therapy in human glioblastoma
Source: EMBO Mol Med. 2019 Feb 27;11(4):e8492. doi: 10.15252/emmm.201708492 (PMC6460351; doi:10.15252/emmm.201708492)
Supplement: Supplementary file 2 — Source Data for Expanded View [file EMMM-11-e8492-s009.zip › 8492-EV-source-data/Source_Data_Figure_EV5.pdf]

**A**

Connexin 26

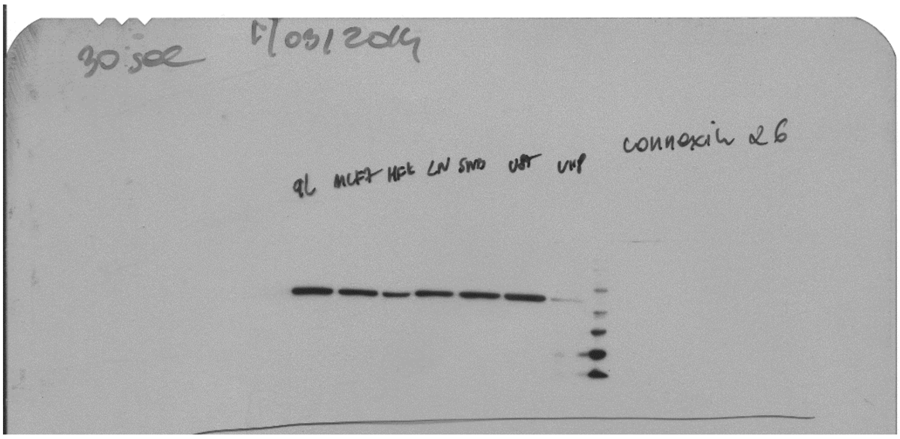


GAPDH

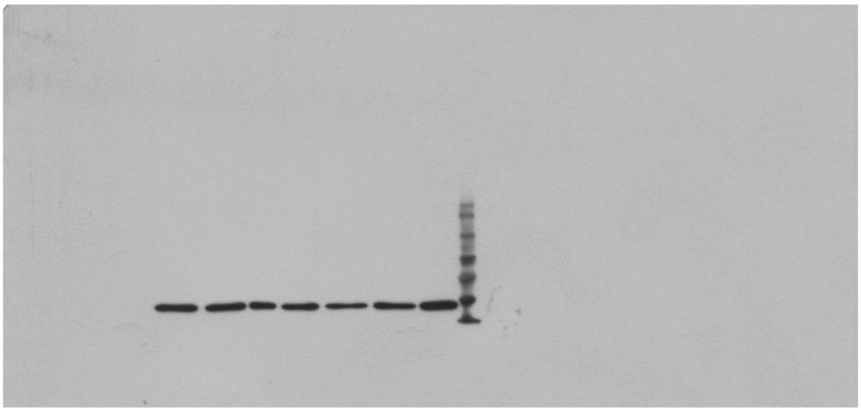

**B**

| | Expression of Connexin 26 normalised to GAPDH | |
|---|---|---|
| **LN229** | 84.22939 | 83.27759 |
| **SNB19** | 91.42857 | 92.52925 |
| **U87** | 85.49528 | 85.69519 |

## Figure EV5- Connexin-26 expression in LN229, U87 and SNB19 cell lines.
