# Supplementary material for: Efficacy of systemic temozolomide‐activated phage‐targeted gene therapy in human glioblastoma
Source: EMBO Mol Med. 2019 Feb 27;11(4):e8492. doi: 10.15252/emmm.201708492 (PMC6460351; doi:10.15252/emmm.201708492)
Supplement: Supplementary file 2 — Source Data for Expanded View [file EMMM-11-e8492-s009.zip › 8492-EV-source-data/Source_Data_Figure_EV1.pdf]

| | RGD4C/AAVP-*Grp78-Luc* | | | | Non-targeted/AAVP-*Grp78-Luc* | | | |
|---|---|---|---|---|---|---|---|---|
| **0** | 37.5 | 7.5 | 24.5 | 33.5 | 5.00 | 8.50 | 4.50 | 5.50 |
| **10** | 77.5 | 117.5 | 80.5 | 44.5 | 40.50 | 44.50 | 37.50 | 10.50 |
| **20** | 84.5 | 157.5 | 54.5 | 150.5 | 20.50 | 27.50 | 17.50 | 7.50 |
| **40** | 527.5 | 574.5 | 533.5 | 563.5 | 25.50 | 57.50 | 14.50 | 14.50 |

**Figure EV1B- Induction of RGD4C/AAVP-*Grp78* by curcumin in primary glioma.**
