# Supplementary material for: Efficacy of systemic temozolomide‐activated phage‐targeted gene therapy in human glioblastoma
Source: EMBO Mol Med. 2019 Feb 27;11(4):e8492. doi: 10.15252/emmm.201708492 (PMC6460351; doi:10.15252/emmm.201708492)
Supplement: Supplementary file 2 — Source Data for Expanded View [file EMMM-11-e8492-s009.zip › 8492-EV-source-data/Source_Data_Figure_EV2.pdf]

A

U87

	Days post intracranial cell implantation									
	9					27				
**non-targeted**	27,10	23,90	24,60	26,50	22,10	22,80	23,70	20,50	25,20	22,60
**RGD4C/AAVP *Grp78-HSVtk***	22,20	27,20	24,30	23,10	23,40	23,40	23,00	23,60	23,20	19,40
**TMZ**	26,90	29,20	24,50	27,50	26,80	25,50	27,00	25,10	27,10	25,30
**RGD4C/AAVP *Grp78-HSVtk* +TMZ**	26,30	24,20	27,20	23,80	25,80	27,30	24,50	27,40	23,50	26,00

B

HSJD-GBM-001

	Days post treatment														
	1					5					12				
**non-targeted**	20,2	21,5	25	23,3		20,3	18,9	24,9	20,3		26.4	23			
**RGD4C/AAVP *Grp78-HSVtk***	22,8	22,2	21	22,9	20,8	18,8	18,9	23,6	21,9	19	21,8	21,9	20,5		
**TMZ**	20,9	22,8	20,6	22		21,7	23,4	20,9	22,9		22,6	24,5	21	23,6	
**RGD4C/AAVP *Grp78-HSVtk* +TMZ**	22	22,3	23,8	22,9	24,3	22,1	20,2	22,2	23	23,2	23,3	21,6	23,6	24,1	

	Days post treatment														
	18					22					26				
**non-targeted**	26,7					26									
**RGD4C/AAVP *Grp78-HSVtk***	22	23,4	23,2			21,3	24,6	22,8			21,4	22,7			
**TMZ**	21,5	25,5	22,7	24		23,5	24,9	24,9	23,6		23,4	22,9	24,3		
**RGD4C/AAVP *Grp78-HSVtk* +TMZ**	24,8	22,8	24,4	24,9		24	23	25	24		24,7	22,4	24,3	24,6	

	Days post treatment				
	38				
**non-targeted**					
**RGD4C/AAVP *Grp78-HSVtk***					
**TMZ**	22,5	24			
**RGD4C/AAVP *Grp78-HSVtk* +TMZ**		22,3	24,7	24,6	

Figure EV2- Toxicity evaluation following treatments of nude mice with intracranial GBM
