# Supplementary material for: Efficacy of systemic temozolomide‐activated phage‐targeted gene therapy in human glioblastoma
Source: EMBO Mol Med. 2019 Feb 27;11(4):e8492. doi: 10.15252/emmm.201708492 (PMC6460351; doi:10.15252/emmm.201708492)
Supplement: Supplementary file 2 — Source Data for Expanded View [file EMMM-11-e8492-s009.zip › 8492-EV-source-data/Source_Data_Figure_EV3.pdf]

A

	Relative LDH Level			
**control**	2.069	2.207	1.61475	2.0041
**$2.5x10^9$**	2.2136	1.76315	1.92775	1.7869
**$1x10^{10}$**	2.05715	2.22170	1.78040	2.3109
**$5x10^{10}$**	1.82000	1.77475	1.90515	1.8431

B

	Percent Weight Change			
**control**	1.578947	1.030928	0.00000	2.127660
**$2.5x10^9$**	8.433735	-0.4975124	-0.5780347	0.0000
**$1x10^{10}$**	4.545455	4.477612	-2.209945	0.000000
**$5x10^{10}$**	-6.666667	6.829268	6.111111	11.111110

Figure EV3- Toxicity study in wild type mice.
