# Supplementary material for: Efficacy of systemic temozolomide‐activated phage‐targeted gene therapy in human glioblastoma
Source: EMBO Mol Med. 2019 Feb 27;11(4):e8492. doi: 10.15252/emmm.201708492 (PMC6460351; doi:10.15252/emmm.201708492)
Supplement: Supplementary file 4 — Source Data for Figure 1 [file EMMM-11-e8492-s002.pdf]

A

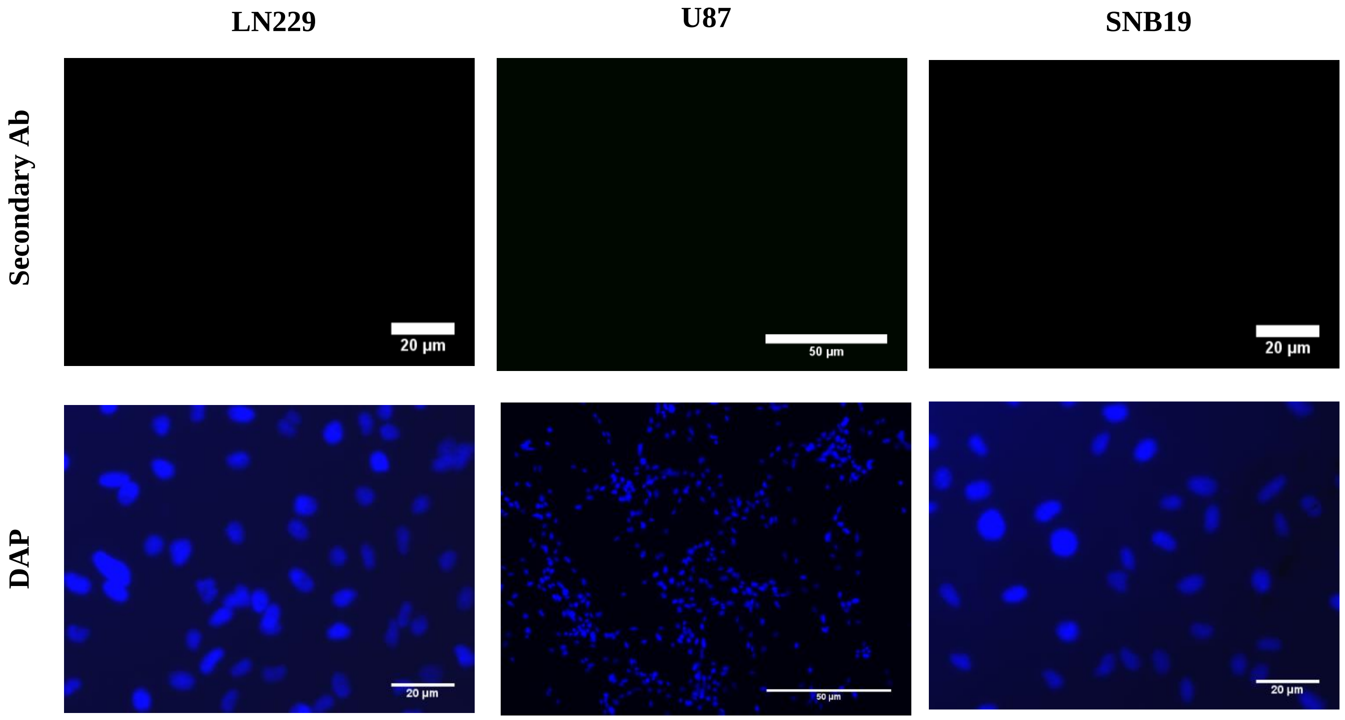

B

LN229

Day	Non-targeted/AAVP-*Luc*			RGD4C/AAVP-*Luc*		
1	9,810553	-12,582210	5,512580	158,418200	4,160052	5,641129
2	-9,360215	4,114339	5,329962	519,960300	668,208200	694,974700
3	7,656897	-7,219358	6,818131	545,872400	686,976100	483,592700
4	42,592070	-12,788640	10,009630	1887,804000	710,291100	1731,185000
5	9,741107	3,820412	3,919520	1682,749000	2155,202000	1953,643000

U87

Day	Non-targeted/AAVP-*Luc*			RGD4C/AAVP-*Luc*		
1	0,8096071	3,694685	5,110786	80,298050	70,233020	80,597710
2	1,639114	3,706106	1,734782	392,806100	671,031400	460,704100
3	11,065350	12,529190	131,006200	672,644000	786,320800	713,909400
4	3,019078	90,721680	40,887590	1256,321000	1247,444000	1471,143000
5	52,691760	107,537900	99,987080	2048,252000	2040,297000	2298,867000

SNB19

Day	Non-targeted/AAVP-*Luc*			RGD4C/AAVP-*Luc*		
1	5,610531	-8,107594	-13,856450	17,761300	11,863760	9,738642
2	12,249370	4,414483	3,977534	49,237390	76,048460	140,122200
3	4,688857	1,532550	1,274226	340,848600	355,245800	302,623900
4	110,614200	42,317300	4,547429	480,337000	435,102100	486,758400
5	2,647971	21,376580	19,482430	541,676000	554,037500	591,498400

C

LN229

Grp78 **GAPDH**

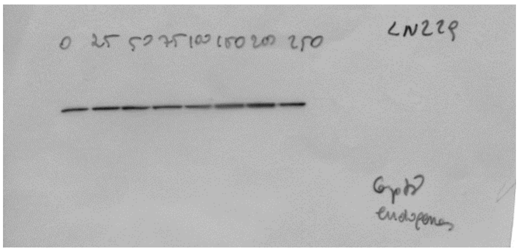

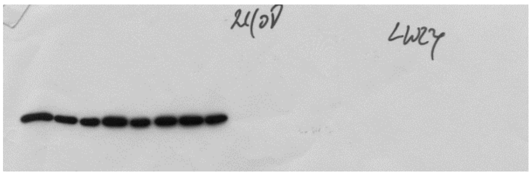

U87

Grp78 **GAPDH**

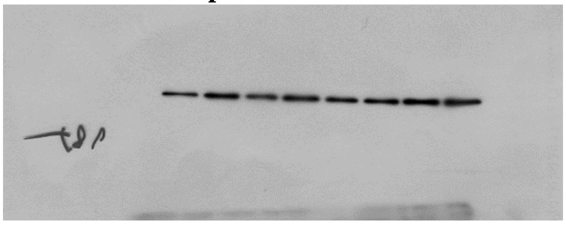

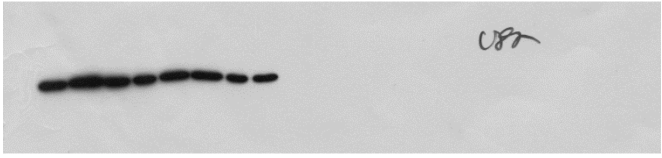

SNB19

Grp78 **GAPDH**

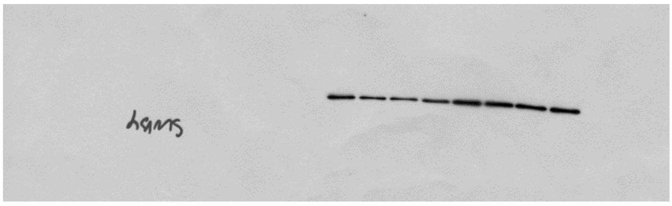

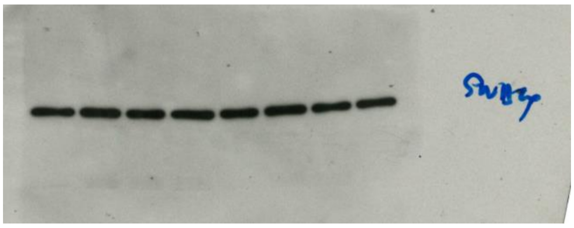

TMZ µM	LN229		U87		SNB19	
**25**	97,193810	89,834420	58,900590	77,534500	69,893460	91,120830
**50**	92,721470	86,165250	69,076350	83,629270	72,013920	91,863350
**75**	110,172800	89,645970	70,786480	74,418030	102,623000	103,452800
**100**	111,825700	101,716900	80,177570	73,617430	118,849600	107,563200
**150**	98,602600	93,688150	108,312500	97,154330	122,837100	100,465600
**200**	106,364600	108,486700	165,770100	147,737000	119,019400	109,240800
**250**	132,246300	152,835300	191,336600	199,615000	123,828600	106,555800

D

LN229

Hours	RGD4C/AAVP-*CMV-Luc*			RGD4C/AAVP-*Grp78-Luc*		
1	2936,939000	8284,805000	2381,161000	33786,030000	205087,500000	28193,120000
2	1490,459000	5849,790000	2723,354000	22705,740000	34377,950000	67805,250000
3	2278,453000	2431,845000	1777,738000	22928,700000	72542,800000	26245,130000
6	37834,570000	12291,290000	63168,800000	58312,180000	20757,850000	37989,520000
9	13318,670000	78208,330000	14056,210000	635976,100000	388628,300000	390275,200000
12	3188,594000	1574,155000	2885,067000	388966,800000	663517,500000	624451,100000
24	20977,640000	44734,360000	23773,860000	422507,800000	619960,400000	654713,200000

U87

Hours	RGD4C/AAVP-*CMV-Luc*			RGD4C/AAVP-*Grp78-Luc*		
1	149905,700000	631020,800000	57083,840000	295628,100000	238193,900000	314469,400000
2	14217,590000	16296,200000	39313,920000	260991,500000	463989,400000	492370,300000
3	37607,300000	366026,200000	370194,800000	282495,000000	272071,300000	570599,000000
6	39922,640000	208657,100000	23686,010000	343280,300000	358137,800000	538212,000000
9	14281,490000	379279,500000	34095,520000	1861823,000000	1386869,000000	1807141,000000
12	106183,500000	10036,190000	68503,700000	1530734,000000	1526206,000000	2235250,000000
24	882060,000000	402890,900000	132820,200000	1857305,000000	2077726,000000	2204814,000000

SNB19

Hours	RGD4C/AAVP-*CMV-Luc*			RGD4C/AAVP-*Grp78-Luc*		
1	416647,700000	414459,600000	339484,300000	413950,600000	457036,000000	476159,100000
2	829918,600000	506631,900000	691322,600000	1202749,000000	473653,700000	1263968,000000
3	211075,900000	223775,000000	257470,700000	462554,000000	620181,700000	633846,100000
6	5331778,000000	371517,000000	375410,800000	2871222,000000	776778,200000	536561,600000
9	592124,400000	742289,100000	876517,100000	1016387,000000	1268375,000000	1623303,000000
12	680612,800000	692208,200000	739553,300000	1046521,000000	1047446,000000	1090348,000000
24	1873496,000000	1375675,000000	1595929,000000	5480756,000000	7543195,000000	7037867,000000

Figure 1- Targeted transduction of human glioblastoma cell lines and induction of RGD4C/AAVP-*Grp78* by TMZ.
