# Supplementary material for: Efficacy of systemic temozolomide‐activated phage‐targeted gene therapy in human glioblastoma
Source: EMBO Mol Med. 2019 Feb 27;11(4):e8492. doi: 10.15252/emmm.201708492 (PMC6460351; doi:10.15252/emmm.201708492)
Supplement: Supplementary file 5 — Source Data for Figure 2 [file EMMM-11-e8492-s003.pdf]

A

LN229

eIF2

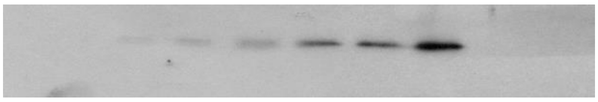

ATF6

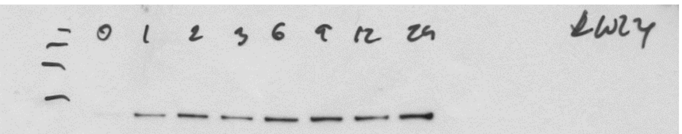

GAPDH

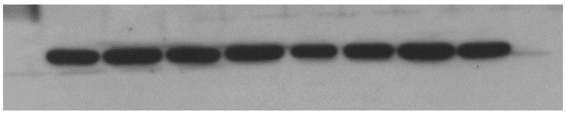

U87

eIF2

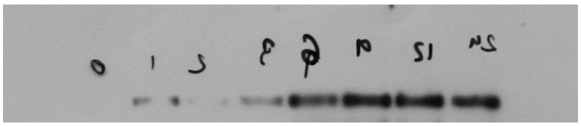

ATF6

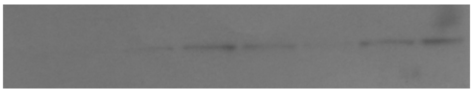

GAPDH

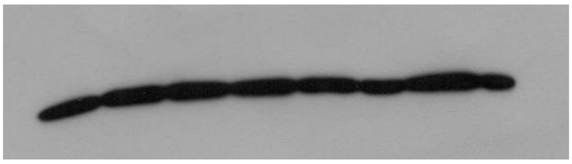

SNB19

eIF2

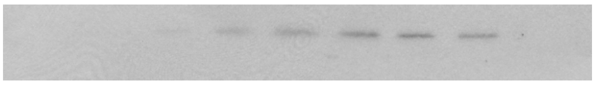

ATF6

GAPDH

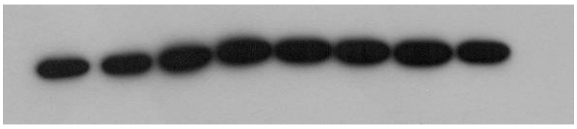

B

LN229

XBP1

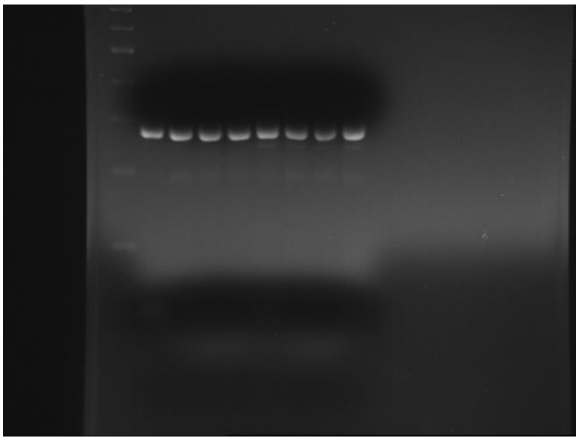

GAPDH

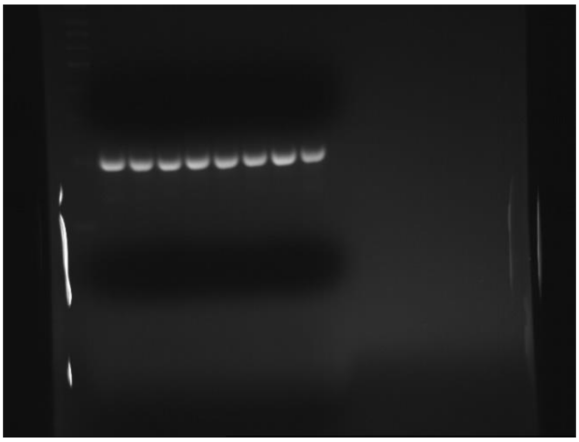

U87

XBP1

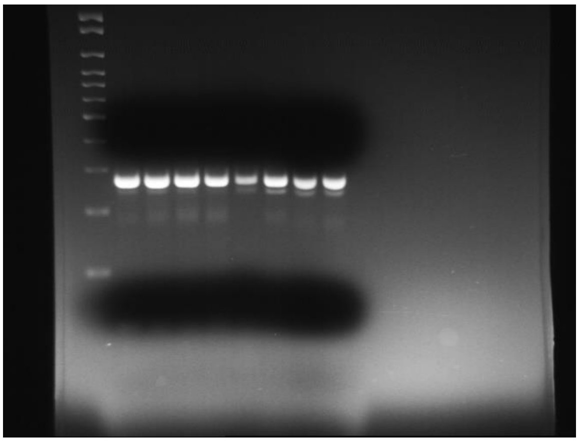

GAPDH

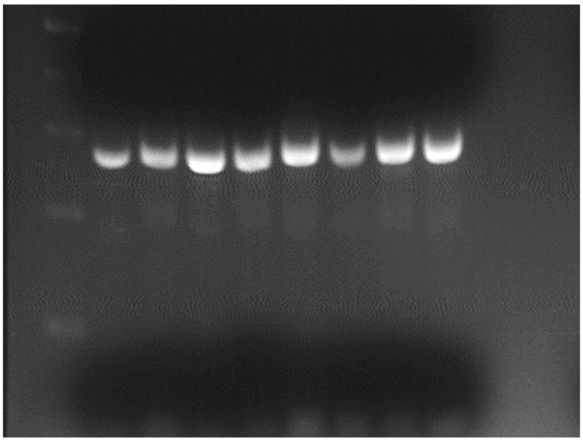

SNB19

XBP1

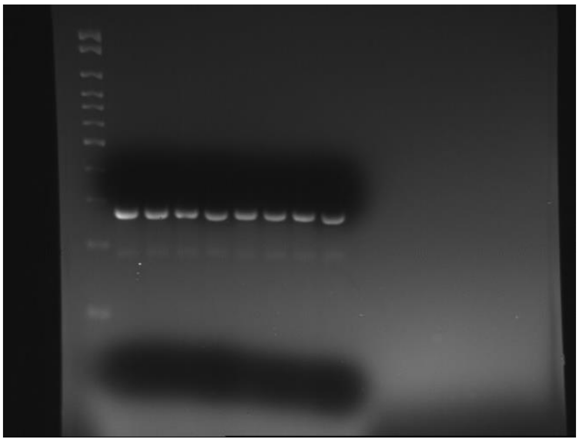

GAPDH

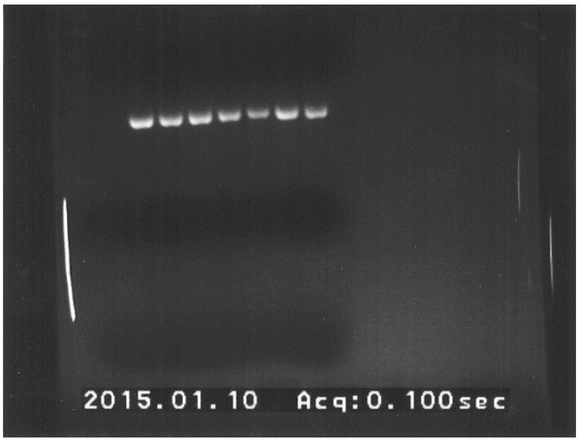

C

LN229

Treatment	RGD4C/AAVP-*CMV-HSVtk*			RGD4C/AAVP-*Grp78-HSVtk*		
**control**	2,65	2,50	2,99	4,40	3,51	3,98
**TMZ**	12,80	10,20	14,70	28,50	26,60	25,70
**GCV**	75,60	74,80	71,33	82,40	85,30	87,00
**TMZ + GCV**	78,80	82,60	81,90	88,20	91,00	90,20

U87

Treatment	RGD4C/AAVP-*CMV-HSVtk*			RGD4C/AAVP-*Grp78-HSVtk*		
**control**	12,00000	13,10000	12,80000	6,95000	7,74000	7,34000
**TMZ**	11,36667	10,26667	10,81667	10,95667	10,75667	10,85667
**GCV**	59,86667	54,46667	57,16667	63,55667	61,55667	62,55667
**TMZ + GCV**	67,06667	62,26667	64,66667	77,45667	74,35667	75,90667

SNB19

Treatment	RGD4C/AAVP-*CMV-HSVtk*			RGD4C/AAVP-*Grp78-HSVtk*		
**control**	4,94	5,26	5,17	9,45	11,20	9,35
**TMZ**	9,84	9,31	9,63	23,90	18,90	21,90
**GCV**	48,90	37,90	44,40	71,20	62,40	65,40
**TMZ + GCV**	59,60	64,10	59,30	82,20	83,20	89,60

Figure 2- TMZ activation of the **UPR pathway and subsequent enhancement of glioblastoma cell killing *in vitro* by the RGD4C/AAVP-*Grp78-HSVtk* and GCV**
