# Supplementary material for: Efficacy of systemic temozolomide‐activated phage‐targeted gene therapy in human glioblastoma
Source: EMBO Mol Med. 2019 Feb 27;11(4):e8492. doi: 10.15252/emmm.201708492 (PMC6460351; doi:10.15252/emmm.201708492)
Supplement: Supplementary file 6 — Source Data for Figure 3 [file EMMM-11-e8492-s004.pdf]

C

	RGD4C/AAVP-*CMV-HSVtk*				Non-targeted			
**Brain Tumor**	12.093210	10.110700	11.763180	12.331310	1.000000	1.000000	1.000000	1.000000
**Brain tissue**	1.526791	1.753509	2.080406	2.133436	1.000000	1.000000	1.000000	1.000000
**Pancreas**	0.6863734	0.6558629	0.3406803	1.443835	1.000000	1.000000	1.000000	1.000000

D

	output				input			
**Brain Tumor**	7360000	6160000	7160000	7500000	50000000000	50000000000	50000000000	50000000000

Figure 3- Systemic targeting of orthotopic glioblastoma with RGD4C/AAVP-*Grp78-HSVtk*.
