# Supplementary material for: Efficacy of systemic temozolomide‐activated phage‐targeted gene therapy in human glioblastoma
Source: EMBO Mol Med. 2019 Feb 27;11(4):e8492. doi: 10.15252/emmm.201708492 (PMC6460351; doi:10.15252/emmm.201708492)
Supplement: Supplementary file 7 — Source Data for Figure 4 [file EMMM-11-e8492-s005.pdf]

B

	Days post intracranial cell implantation									
	9					27				
**non-targeted**	1,3870	3,6140	1,6020	0,670500	1,2410	21,3300	6,3510	4,7630	20,250000	22,4800
**RGD4C/AAVP *Grp78-HSVtk***	6,8780	3,6040	2,8230	0,729400	1,8610	6,7690	8,6780	6,6900	28,780000	28,7800
**TMZ**	0,4734	0,2085	0,8918	0,408500	0,7929	7,3300	1,9860	1,1360	3,884000	5,5140
**RGD4C/AAVP *Grp78-HSVtk* +TMZ**	3,2800	6,3510	4,3330	0,652000	0,5072	0,7777	2,2350	0,7837	1,859000	2,1840

C

	Days post intracranial cell implantation									
	9					27				
**non-targeted**	100,	100,	100,	100,	100,	1252,569	372,952	279,6993	1189,148	1320,101
**RGD4C/AAVP *Grp78-HSVtk***	100,	100,	100,	100,	100,	212,9232	272,972	210,4382	905,2933	905,2933
**TMZ**	100,	100,	100,	100,	100,	1320,673	357,825	204,6773	699,7946	993,4777
**RGD4C/AAVP *Grp78-HSVtk* +TMZ**	100,	100,	100,	100,	100,	25,71215	73,89309	25,91052	61,46186	72,20694

Figure 4- Therapeutic response of orthotopic U87 glioblastoma to combination of TMZ with RGD4C/AAVP-*Grp78-HSVtk* plus GCV
