# Supplementary material for: Efficacy of systemic temozolomide‐activated phage‐targeted gene therapy in human glioblastoma
Source: EMBO Mol Med. 2019 Feb 27;11(4):e8492. doi: 10.15252/emmm.201708492 (PMC6460351; doi:10.15252/emmm.201708492)
Supplement: Supplementary file 8 — Source Data for Figure 6 [file EMMM-11-e8492-s006.pdf]

C

HSJD-GBM-001

Day	Non-targeted/AAVP-*Luc*			RGD4C/AAVP-*Luc*		
2	293	273	266	3570	5230	773
3	280	306	296	21700	20400	10900
4	343	336	326	151000	115000	128000
5	640	443	716	149000	174000	209000
6	1410	1140	950	377000	444000	276000
7	2080	953	1180	551000	322000	664000
8	3330	1740	1200	565000	622000	520000

G26

Day	Non-targeted/AAVP-*Luc*							
**2**	490	520	486	556	556	450	410	516
**3**	586	530	623	576	636	610	470	610
**4**	553	500	500	436	760	526	450	596
**5**	380	420	426	436	506	473	416	340
**6**	446	490	433	443	373	436	410	380
**7**	396	353	360	416	460	360	320	296
**8**	443	396	366	300	5050	516	340	403

Day	RGD4C/AAVP-*Luc*							
**2**	580	1160	2650	1210	1260	623	656	766
**3**	2780	3560	6380	2030	8650	9800	7600	3860
**4**	5520	6880	9320	3000	8100	8570	6700	7480
**5**	16400	18100	19700	8270	19800	16100	10800	20900
**6**	28700	21900	44100	24400	22200	21100	22400	31100
**7**	49200	33900	55800	35100	28100	32500	39700	59500
**8**	69900	45800	82100	53500	53500	61300	68400	143000

Figure 6- Characterization of the human primary GBM, then analysis of targeted gene delivery
