# Supplementary material for: Efficacy of systemic temozolomide‐activated phage‐targeted gene therapy in human glioblastoma
Source: EMBO Mol Med. 2019 Feb 27;11(4):e8492. doi: 10.15252/emmm.201708492 (PMC6460351; doi:10.15252/emmm.201708492)
Supplement: Supplementary file 9 — Source Data for Figure 7 [file EMMM-11-e8492-s007.pdf]

B

Astrocytes

Day	Non-targeted/AAVP-*Luc*				RGD4C/AAVP-*Luc*			
**2**	223	236	186	223	220	263	236	210
**3**	250	223	316	273	210	290	256	253
**4**	293	350	333	326	383	396	416	373
**5**	466	300	250	276	253	246	263	260
**6**	218	204	217	231	184	210	211	177
**7**	383	310	290	273	330	270	293	313
**8**	356	266	226	250	193	233	226	233

TMZ	RGD4C/AAVP-*Luc*			
0.	193	233	226	233
35.	193	230	253	180
70.	220	220	220	236
100.	173	226	266	246
150.	190	190	180	223

Skin

Day	Non-targeted/AAVP-*Luc*				RGD4C/AAVP-*Luc*			
**2**	236	336	350	336	296	283	306	320
**3**	333	323	363	346	336	356	443	400
**4**	410	296	380	273	426	280	303	293
**5**	346	366	413	436	363	410	416	436
**6**	593	606	503	516	460	400	440	450
**7**	490	520	516	446	556	566	520	523
**8**	373	380	370	403	386	403	373	400

TMZ	RGD4C/AAVP-*Luc*			
0.	386	403	373	400
35.	360	410	383	373
70.	366	453	446	476
100.	336	356	393	330
150.	323	363	380	373

Lung

Day	Non-targeted/AAVP-*Luc*				RGD4C/AAVP-*Luc*			
**2**	506	413	423	476	410	460	396	463
**3**	350	396	360	370	403	296	320	336
**4**	350	356	360	380	360	326	343	406
**5**	283	220	253	283	216	240	216	310
**6**	356	326	310	426	283	286	246	323
**7**	340	340	353	373	283	386	380	356
**8**	423	346	356	316	406	346	346	320

TMZ	RGD4C/AAVP-*Luc*			
0.	406	346	346	320
35.	406	336	353	316
70.	380	336	340	356
100.	410	290	306	306
150.	436	400	373	370

Figure 7- Integrin expression in human primary normal cells, then analysis of targeted gene delivery.
