# Supplementary material for: Efficacy of systemic temozolomide‐activated phage‐targeted gene therapy in human glioblastoma
Source: EMBO Mol Med. 2019 Feb 27;11(4):e8492. doi: 10.15252/emmm.201708492 (PMC6460351; doi:10.15252/emmm.201708492)
Supplement: Supplementary file 10 — Source Data for Figure 9 [file EMMM-11-e8492-s008.pdf]

C

	Days post intracranial cell implantation									
	**10**					**34**				
**non-targeted**	100,	100,	100,	100,	100,	45.36823	29.44786	1074.673	1124.075	43.61217
**RGD4C/AAVP *Grp78-HSVtk***	100,	100,	100,	100,	100,	180.6431	54.78537	140.1765	42.87887	125.4718
**TMZ**	100,	100,	100,	100,	100,	293.3208	15.71775	2.491297	576.4139	2.668833
**RGD4C/AAVP *Grp78-HSVtk* +TMZ**	100,	100,	100,	100,	100,	3.238107	44.48055	2.91391	13.31329	2.444983

E

	Days post intracranial cell implantation											
	**10**						**34**					
**non-targeted**	17.9	18.8	18.3	17.7	19.6	20.6	15.8	20.3		16.5		22.6
**RGD4C/AAVP *Grp78-HSVtk***	19.3	18.1	18.1	17.4	19.4	18.9		19.8	18.5	18.3	20.9	16.3
**TMZ**	18.2	19.4	17.5	19.7	20.5	20.8	19.4	20.1	18.8	16.8	21.7	23.5
**RGD4C/AAVP *Grp78-HSVtk* +TMZ**	18.7	19.1	19.1	19.8	17.6	20.8	20.9	21.3	20.2	20.9	18.2	20.5

Figure 9- RGD4C/AAVP-*Grp78-HSVtk* plus GCV gene therapy and combination with TMZ against orthotopic murine GL261 GBM
